# Fault Feature Extraction and Diagnosis of Rolling Bearings Based on Enhanced Complementary Empirical Mode Decomposition with Adaptive Noise and Statistical Time-Domain Features

**DOI:** 10.3390/s19184047

**Published:** 2019-09-19

**Authors:** Liwei Zhan, Fang Ma, Jingjing Zhang, Chengwei Li, Zhenghui Li, Tingjian Wang

**Affiliations:** 1 Aero Engine Corporation of China Harbin Bearing Co., LTD, Harbin 150500, China; fangma666@163.com (F.M.); zhangjingjing12311@163.com (J.Z.); zhenghuili90@163.com (Z.L.); 2 School of Electrical Engineering and Automation, Harbin Institute of Technology, Harbin 150001, China; 3 College of Mechanical Engineering, Tianjin University of Technology and Education, Tianjin 300222, China; tjwang@hit.edu.cn

**Keywords:** fault diagnosis, computational cost, ensemble trails, amplitude of added white noise, variable speed

## Abstract

In this paper, a novel method is proposed to enhance the accuracy of fault diagnosis for rolling bearings. First, an enhanced complementary empirical mode decomposition with adaptive noise (ECEEMDAN) method is proposed by determining two critical parameters, namely the amplitude of added white noise (AAWN) and the ensemble trails (ET). By introducing the concept of decomposition level, the optimal AAWN can be determined by judging the mutation of mutual information (MI) between adjacent intrinsic mode functions (IMFs). Furthermore, the ET is fixed at two to reduce the computational cost. This method can avoid disturbance of the spurious mode in the signal decomposition and increase computational speed. Enhanced CEEMDAN demonstrates a more significant improvement than that of the traditional CEEMDAN. Vibration signals can be decomposed into a set of IMFs using enhanced CEEMDAN. Some IMFs, which are named intrinsic information modes (IIMs), effectively reflect the vibration characteristic. The evaluated comprehensive factor (CF), which combines the shape, crest and impulse factors, as well as the kurtosis, skewness, and latitude factor, is developed to identify the IIM. CF can retain the advantage of a single factor and make up corresponding drawbacks. Experiment results, especially for the extraction of bearing fault under variable speed, illustrate the superiority of the proposed method for the fault diagnosis of rolling bearings over other methods.

## 1. Introduction

The rolling bearing is an important part of rotating machinery. Fault diagnosis is significant to ensure normal machinery operation [1,2,3]. A diagnosis method which concentrates on the time domain, frequency domain, and time-frequency domain has been proposed to minimize the interference and extract the fault feature due to interferences from heavy background noise and other unsteady operation states [4,5]. However, the time and frequency domain methods are unsuitable in fault diagnosis in the case of non-stationary and non-linear vibration signals [6]. Time-frequency domain methods, such as wavelet transform [7] and Wigner–Ville distribution [8], have been used to extract the fault feature of rolling bearings. However, these methods cannot obtain the ideal time-frequency resolution subject to inherent cross-interference items [9] and Heisenberg’s uncertainty principle [10]. Hence, developing a signal processing method to identify the fault feature of rolling bearings is necessary. 

Originally, empirical mode decomposition (EMD) [11] is an adaptive data-driven method to process non-linear and non-stationary signals. This technology has been applied in various fields, such as the fault diagnosis of rotational machinery [12,13], signal filtering [14,15], and biomedical signal processing [16]. However, the decomposed IMFs may include discrete scales in one mode, or alike scales in different modes. This annoying problem, termed as ‘mode mixing’, limits the application of EMD. The ensemble EMD (EEMD) [17] was developed by adding Gaussian white noise into the original signal to solve the problem of mode mixing. IMFs can be obtained by averaging the ensemble copies of the original signal. Although the EEMD can reduce ‘mode mixing’, this approach also introduces a new problem. The IMFs contain the residue white noise, and the different realizations of signal and noise may produce spurious modes. Consequently, complementary EEMD (CEEMD) [18] was introduced to eliminate the residue noise by adding a pair of white noise (positive and negative) into the signal. Although the added white noise can be partly eliminated, the decomposed IMFs still contain the spurious modes. CEEMD with adaptive noise (CEEMDAN) [19] was then proposed to solve the problems of residual noise and the spurious mode. However, the improved versions of EMD (EEMD, CEEMD, and CEEMDAN) involve two critical parameters, namely the amplitude of added white noise (AAWN) and the number of ensemble trials (ET). If the AAWN is small, then eliminating mode mixing in the EMD will be difficult, however, if the AAWN is large, sufficient ETs are needed to eliminate the residue noise. Similarly, the program execution requires a high computational cost. Hence, determining AAWN and ET is challenging. Huang et al. [17] selected two critical parameters using the empirical method, however, the method is unsuitable to decompose signals for any case. Zhang et al. [20] proposed correlation coefficients between IMFs and the original signal to determine the suitable ET. Niazy et al. [21] employed the relative root mean square error to find the appropriate AAWN. However, this method does not select the noisy level. Lei et al. [22] proposed the adaptive EEMD method, which focuses on the selection of AAWN and the ET, to improve the decomposition performance. However, the determination of AAWN and ET is still conducted using empirical methods. Thus, developing a method to determine the proper parameters (AAWN and ET) is necessary. 

Moreover, the entire IMFs do not effectively reflect the vibrational characteristic of the bearing fault. The intrinsic information of the vibration signal concentrates on an IMF or several IMFs, which are known as intrinsic information modes (IIMs). Recently, several studies have reported the identification of IIMs. For example, kurtosis [23] is sensitive to bearing vibration signals. However, when the fault becomes severe, it is not consistent with the fault development. Using the correlation coefficient [24] and Kullback–Leibler divergence [25] does not work well for fault identification. Sample entropy (SE) [26], approximate entropy (AE) [27], and fuzzy entropy (FE) [28] have been recently introduced into the health monitoring domain. However, SE is suddenly changed and non-continuous at the boundary. AE has the drawback of heavily reliant on the dimensions of the given data. FE employs the membership function, which is difficult to determine. Hence, introducing a method to identify IIMs is urgent.

To solve the aforementioned problem, this paper first proposes the enhanced CEEMDAN (ECEEMDAN) method. In this method, the ET is fixed at two trails to reduce the computational cost. By introducing the concept of decomposition level, the optimal AAWN can then be determined by judging the mutation of mutual information (MI) between adjacent IMFs at different decomposition levels. Finally, the ECEEMDAN suppresses the interference of the spurious mode, and the decomposed IMF has physical meaning. The statistical methods, such as the shape, crest, and impulse factors, as well as the kurtosis, skewness, and latitude factor, are given the same weight to constitute a complete comprehensive factor (CF) to determine the IIM. Through simulation and the analysis of real signals the effectiveness of the proposed method is verified. The results indicate that the proposed method performs well for the fault diagnosis of rolling bearings.

The remainder of this paper is organized as follows. Section 2 introduces the related works to illustrate the principles of existing algorithms. Section 3 describes the proposed works. Section 4 evaluates the effectiveness of the proposed method for fault feature extraction. Finally, Section 5 presents the conclusion.

## 2. Relevant Works

### 2.1. CEEMDAN Algorithm

Although the EEMD [17] can solve mode mixing in EMD [11], this approach presents two drawbacks, namely the residual noise and spurious mode. Consequently, CEEMDAN [19] is introduced to alleviate these issues. This process is carried out as follows:

1. Generate the noisy signal $x^{i}$, $x^{i}=x+\omega_{0}\xi^{i}\left(i=1,\cdots ,N\right)$, where $\xi^{i}$ is white noise with unit variance, $\omega_{0}$ is coefficient of added white noise, and x is the discrete signal.
2. Extract the first IMF ($c_{1}^{i}$) in each noisy signal $x^{i}$ decomposition by EMD.
3. Obtain the first IMF ($c_{1}$) by taking the average of each $c_{1}^{i}$.
  (1) $$c_{1}=\frac{1}{N}\sum_{i=1}^{N}c_{1}^{i}$$
4. Acquire the first residual: $r_{1}=x-c_{1}$.
5. Apply EMD to decompose $r_{1}+w_{1}E_{1}\left(\varepsilon^{i}\right)$ and extract the first IMF to obtain the second decomposed IMF ($c_{2}$). $w_{1}$ is the coefficient of added white noise for this stage, and operator $E_{m}\left(\cdot \right)$ is the m-th IMF by EMD decomposition.
  (2) $$c_{2}=\frac{1}{N}\sum_{i=1}^{N}E_{1}\left(r_{1}+w_{1}E_{1}\left(\varepsilon^{i}\right)\right)$$
6. Compute the m-th residual mode (m=2,…,K), and extract the first IMF to generate the (m+1)-th decomposed IMF by Equation (3).
  (3) $$c_{m+1}=\frac{1}{N}\sum_{i=1}^{N}E_{1}\left(r_{m}+w_{m}E_{m}\left(\varepsilon^{i}\right)\right)$$
7. Repeat the above steps until the residual R contains less than two extrema.
  (4) $$R=x-\sum_{m=1}^{K}c_{k}$$

The discrete signal x can be written as:

(5) $$x=\sum_{m=1}^{K}c_{k}+R$$

where K is the number of IMFs.

### 2.2. Mutual Information 

Information theory, which originally deals with communication problems, was developed by Shannon [29]. In this study, the key measure was entropy. Afterward, information theory was also applied to feature extraction [30,31,32].

Let $x_{i}$ be a discrete series with n data length. $p\left(x_{i}\right)$ is the probability mass function for discrete series $x_{i}$. The entropy H(X) is defined as:

(6) $$H\left(X\right)=-\sum_{i=1}^{n}p\left(x_{i}\right)\log p\left(x_{i}\right)$$

The joint entropy can be expressed as:

(7) $$H\left(X,Y\right)=-\sum_{i=1}^{n}\sum_{j=1}^{m}p\left(x_{i},y_{j}\right)\log p\left(x_{i},y_{j}\right)$$

where $p\left(x_{i},y_{j}\right)$ is the joint probability distribution of discrete series $x_{i}$ and $y_{j}$. The conditional entropy H(X|Y) for discrete series $x_{i}$ is defined as:

(8) $$H\left(X|Y\right)=-\sum_{i=1}^{n}\sum_{j=1}^{m}p\left(x_{i},y_{j}\right)\log p\left(x_{i}|y_{j}\right)$$

where $p\left(x_{i}|y_{j}\right)$ is the posterior probabilities of X given Y, which can be rewritten as:

(9) H(X|Y)=H(X,Y)−H(Y)

Thus, the MI [33] is introduced to quantify the shared information by discrete series $x_{j}$ and $y_{j}$:

(10) $$I\left(X;Y\right)=\sum_{i=1}^{n}\sum_{j=1}^{m}p\left(x_{i},y_{j}\right)\log\frac{p\left(x_{i}|y_{j}\right)}{p\left(x_{i}\right)}$$

Moreover, the entropy and MI can be related (Equation (11)):

(11) I(X;Y)=H(X)−H(X|Y)

By combining Equations (9) and (11), the MI is re-expressed as:

(12) I(X;Y)=H(X)+H(Y)−H(X,Y)

## 3. Proposed Works

### 3.1. ECEEMDAN 

Using the traditional CEEMDAN method, a discrete signal x can be decomposed into the following modes $c_{i}$(i=1,…,N):

(13) $$\mathrm{CEEMDAN}\left(x,\mathrm{AAWN},\mathrm{ET}\right)=\sum_{i=1}^{N}c_{i}+R$$

Equation (13) shows that the decomposition effect is related to two parameters, namely AAWN and ET. Traditionally, the AAWN is 0.2–0.5 times the strand deviation of the signal, and the ET is fixed at several hundred ensemble trails [17]. However, the specified guidelines for the selection of the two critical parameters are lacking. To solve this problem, first, ET is fixed at two ensemble trails. If the ET is fixed at one trail, then the process of IMF decomposition using CEEMDAN is the same as the EMD method, preventing the elimination of mode mixing. If the ET is bigger than the two trails, then the computational speed will decrease. Hence, the optimal ET is two trails. Then, the different AAWN is defined using the following expression:

(14) $${\mathrm{AAWN}}_{i}=L_{i}\sigma \;\left(i=1,\ldots ,n\right)$$

where σ is the strand deviation of signal and $L_{i}$ is the decomposition level. Thus, Equation (13) can be rewritten as:

(15) $$\mathrm{CEEMDAN}\left(x,\sigma L_{i},2\right)=\sum_{j=1}^{N_{i}}c_{ij}+R$$

If the decomposition level $L_{i}$ is determined, then the two critical parameters are also identified. MI is introduced in this study to determine the optimal decomposition level ($L_{opt}$), and $\left(L_{1},\ldots ,L_{N}\right)$ is the defined decomposition level. A set of IMFs ($c_{ij}$($j=1,\ldots ,N_{i}$)) can be obtained for each decomposition level $L_{i}$ using CEEMDAN. Then, the MI between adjacent IMFs ($c_{ij}$ and $c_{i\left(j+1\right)}$) is calculated. The MI for each $L_{i}$ can be expressed as:

(16) $$\left[\begin{array}{c} \begin{array}{cccc} {\mathrm{MI}}_{11} & {\mathrm{MI}}_{12} & \cdots & {\mathrm{MI}}_{1\left(N_{1}-2\right)} \end{array}\\ \begin{array}{cccc} {\mathrm{MI}}_{21} & {\mathrm{MI}}_{22} & \cdots & {\mathrm{MI}}_{2\left(N_{2}-2\right)} \end{array}\\ \vdots \\ \begin{array}{cccc} {\mathrm{MI}}_{N1} & {\mathrm{MI}}_{N2} & \cdots & {\mathrm{MI}}_{N\left(N_{i}-2\right)} \end{array} \end{array}\right]=\mathrm{MI}\left[\begin{array}{cccc} \left(c_{11},c_{12}\right) & \left(c_{12},\;c_{13}\right) & \cdots & \left(c_{1\left(N_{1}-2\right)},\;c_{1\left(N_{1}-1\right)}\right)\\ \left(c_{21},\;c_{22}\right) & \left(c_{22},\;c_{23}\right) & \cdots & \left(c_{2\left(N_{2}-2\right)},\;c_{2\left(N_{2}-1\right)}\right)\\ \vdots & \vdots & \vdots & \vdots \\ \left(c_{N1},\;c_{N2}\right) & \left(c_{N2},\;c_{N3}\right) & \cdots & \left(c_{N\left(N_{i}-2\right)},\;c_{N\left(N_{i}-1\right)}\right) \end{array}\right]$$

**Figure 1:**
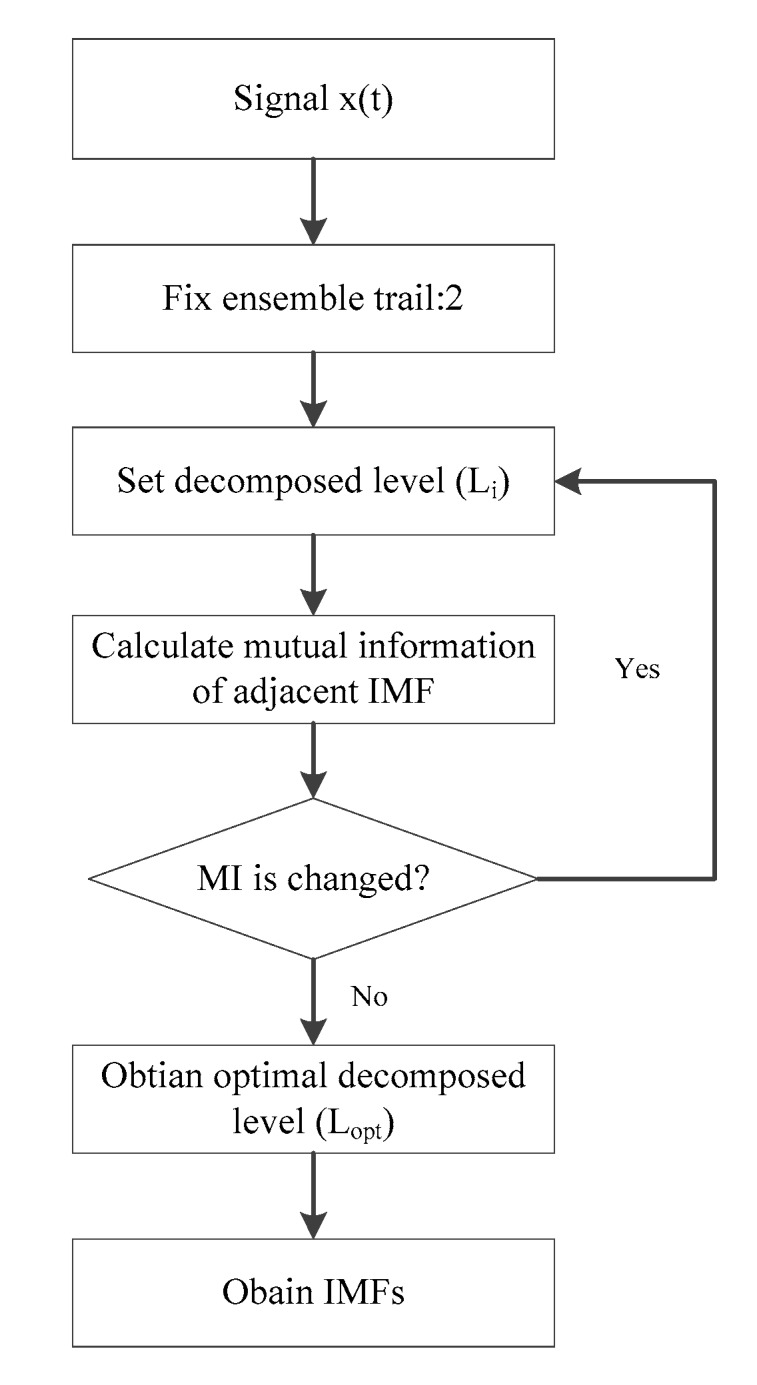
Flowchart of enhanced complementary empirical mode decomposition with adaptive noise (ECEEMDAN).

If no interference of spurious modes is observed, then the MI of corresponding IMF is almost the same under each decomposed level (Equation (17)):

(17) $$\begin{array}{l} {\mathrm{MI}}_{11}\approx {\mathrm{MI}}_{12}\approx \ldots \approx {\mathrm{MI}}_{1\left(M-1\right)}\approx {\mathrm{MI}}_{1M}\\ {\mathrm{MI}}_{21}\approx {\mathrm{MI}}_{22}\approx \ldots \approx {\mathrm{MI}}_{2\left(M-1\right)}\approx {\mathrm{MI}}_{2M}\\ \;\vdots \\ {\mathrm{MI}}_{\left(i-1\right)1}\approx {\mathrm{MI}}_{\left(i-1\right)2}\approx \ldots \approx {\mathrm{MI}}_{\left(i-1\right)\left(M-1\right)}\approx {\mathrm{MI}}_{\left(i-1\right)M}\\ {\mathrm{MI}}_{i1}\approx {\mathrm{MI}}_{i2}\approx \ldots \approx {\mathrm{MI}}_{i\left(M-1\right)}\approx {\mathrm{MI}}_{iM} \end{array}$$

where M is the order of the decomposition level. Once the spurious modes are available in IMFs, the relationship between adjacent IMFs will be broken, indicating that the unsuitability of Equation (17). Hence, the optimal $L_{opt}$ can be determined by judging the change in MI. The Figure 1 presents the flowchart of ECEEMDAN algorithm.

### 3.2. Evaluation Methodology of Decomposing Effect

A signal x can be decomposed into a set of IMFs by ECEEMDAN. Ideally, each IMF has an independent frequency component, and any two IMFs are mutually orthogonal [34]. This means that the scalar product ($\langle c_{i}\cdot c_{j}\rangle \;\left(i\neq j\right)$) should be zero. However, the orthogonality, which means that the product $\langle c_{i}\cdot c_{j}\rangle \;\left(i\neq j\right)$ is not zero, will be broken due to the interaction between the added white noise and the signal. Thus, this paper introduces the concept of an orthogonality index (OI) to evaluate the decomposing effect.

(1) Equation (5) is squared.
  (18) $$x^{2}=\sum_{i=1}^{K+1}c_{i}^{2}+2\sum_{i=1}^{K+1}\sum_{k=1}^{K+1}c_{i}c_{k}$$
(2) Equation (18) is normalized.
  (19) $$1=\frac{\sum_{i=1}^{K+1}c_{i}^{2}}{x^{2}}+\frac{2\sum_{i=1}^{K+1}\sum_{k=1}^{K+1}c_{i}c_{k}}{x^{2}}$$
(3) Equation (19) is reorganized and quantified. The OI is defined as:
  (20) $$\mathrm{OI}=\frac{\sum_{i=1}^{K+1}\sum_{k=1}^{K+1}c_{i}c_{k}}{x^{2}}=\frac{1}{2}\left(1-\frac{\sum_{i=1}^{K+1}c_{i}^{2}}{x^{2}}\right)$$

A small OI results in an improved decomposition effect, and vice versa. Moreover, the decomposition number and computational cost are considered to be the evaluation methodology. The two indexes can reflect the interference situation of spurious mode and computational speed.

### 3.3. Simulation Signal Analysis

Some typical signals, which include pure (noise-free) signals, noisy signals, and simulated vibration signals, were decomposed to verify the effectiveness of ECEEMDAN. Meanwhile, the decomposition effect of traditional methods (EEMD, CEEMD and CEEMDAN) was compared with the ECEEMDAN method.

#### 3.3.1. Free-Noise Signal 

**Figure 2:**
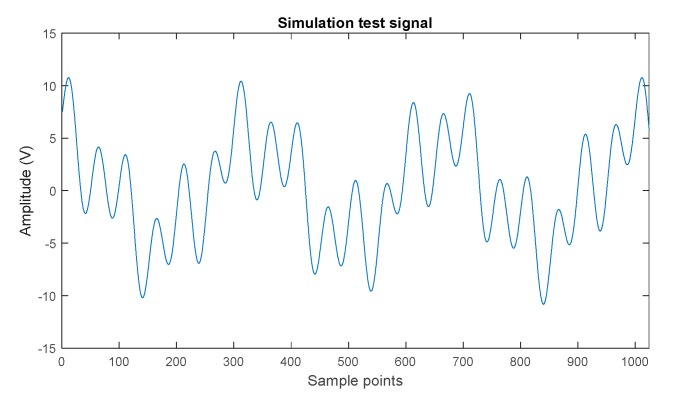
Test simulation signal with the specified frequency component.

In this section, ECEEMDAN is used to decompose the simulation signal, which contains three different frequency components:

(21) $$\{\begin{array}{c} x_{1}\left(t\right)=a_{1}\cos\left(2\pi f_{1}t\right)\\ x_{2}\left(t\right)=a_{2}\cos\left(2\pi f_{2}t\right)\\ x_{3}\left(t\right)=a_{3}\sin\left(2\pi f_{3}t\right) \end{array}$$

(22) $$x\left(t\right)=x_{1}\left(t\right)+x_{2}\left(t\right)+x_{3}\left(t\right)$$

where the amplitudes $a_{1}$, $a_{2}$, and $a_{3}$ were 4, 2.5, and 5, respectively, and the frequencies $f_{1}$, $f_{2}$, and $f_{3}$ were 20 Hz, 10 Hz, and 3 Hz, respectively. The sample frequency ($f_{s}$) was 1 kHz. The data length was 1024. Figure 2 presents the simulation signal.

**Figure 3:**
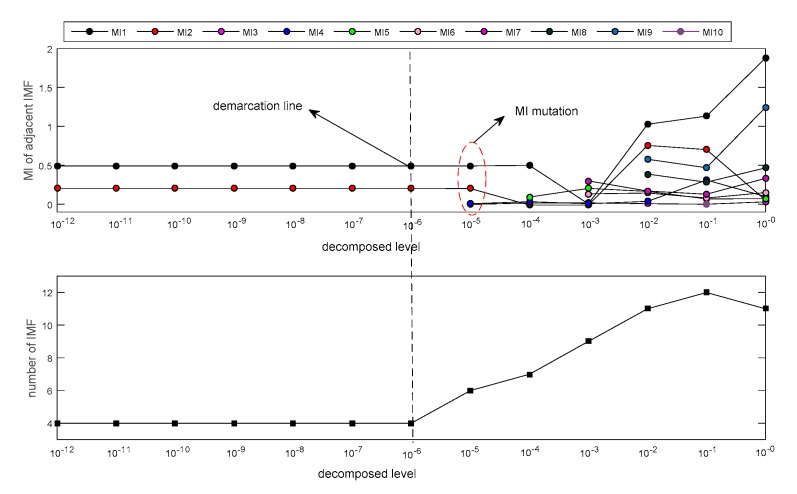
Decomposition results for the simulation signal (SSC) signal under different decomposition levels.

First, ECEEMDAN was used to decompose the simulation signal (SSC). Figure 3 shows the MI of adjacent IMFs under each decomposition level. The decomposition level in this study was set from 10^−12^ to 10^−1^, with a step of 10^1^. The results showed that the MI and decomposition number were the same when the decomposed levels were between 10^−12^ and 10^−6^. However, when decomposition levels were higher than 10^−6^, MI mutation occurred (shown as the red ellipse in Figure 3). This finding indicates that a low decomposition level results in an improved decomposition effect. The decomposed level (L = 10^−6^) was considered as the optimal level in this study.

**Figure 4:**
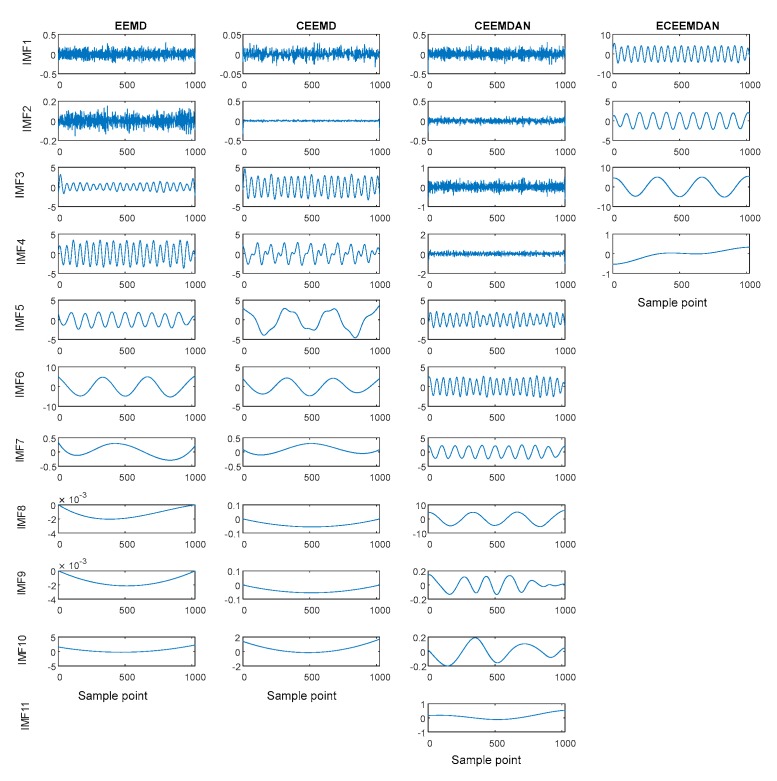
Comparison of decomposing effects of empirical mode decomposition (EMD) improved versions with ECEEMDAN.

Meanwhile, improved EMD versions (EEMD, CEEMD and CEEMDAN) were also used to decompose the SSC (here, the ET and AAWN were fixed at 200 and 0.2, respectively). Figure 4 shows the comparison of ECEEMDAN with the EMD improved versions. The results show three IMFs and one residual in the ECEEMDAN method, whereas the number of IMFs using the EMD improved versions are all more than four.

**Figure 5:**
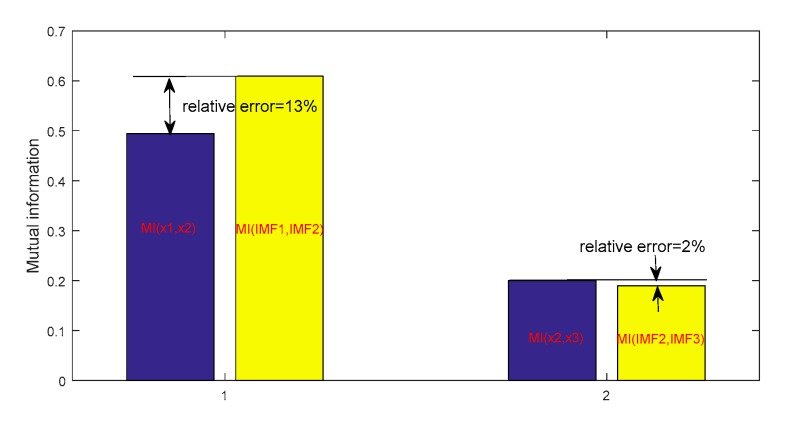
Mutual information (MI) comparison of information component of original signal and corresponding intrinsic mode functions (IMFs).

Meanwhile, the MI of the adjacent sub-signal ($x_{1}\left(t\right)\;\mathrm{and}\;x_{2}\left(t\right),x_{2}\left(t\right)\;\mathrm{and}\;x_{3}\left(t\right)$) in the original signal was also compared with the decomposed IMFs (IMF1 and IMF2, IMF2 and IMF3) using the ECEEMDAN method (Figure 5), and the relative errors were 13% and 2%, respectively. These results illustrate that the IMFs, including the information component, were also almost the same as that of the original signal. Moreover, the spurious modes exist in IMFs using improved EMD versions.

Moreover, the computational cost and OI were compared when using the improved EMD versions (Table 1). The results show that the computational cost and OI using the ECEEMDAN method are smaller than that of the improved EMD versions. Hence, the decomposition effect for the proposed method is superior than that of the traditional methods (EEMD, CEEMD, and CEEMDAN).

#### 3.3.2. Noisy Signal 

**Figure 6:**
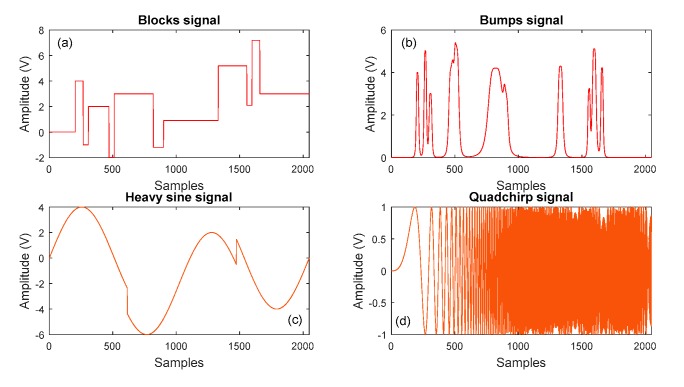
Simulation of the noisy signal.

In this section, the noisy signals, which were ‘blocks’, ‘bumps’, ‘heavy sine waves’ and ‘quadchirps’, were decomposed using ECEEMDAN to prove the performance of the decomposition effect under the effect of noise interference. Here, the data length was 2048. Figure 6 plots the noisy signals.

**Figure 7:**
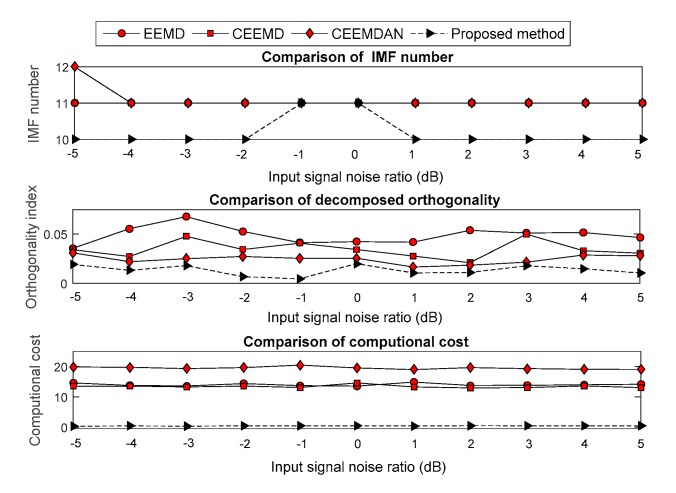
Comparison of the decomposition effect for the ‘blocks’ signal.

**Figure 8:**
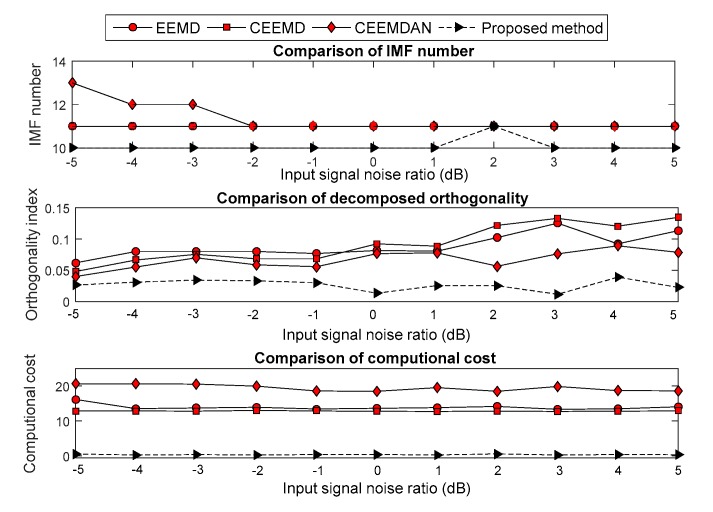
Comparison of the decomposition effect for the ‘bumps’ signal.

**Figure 9:**
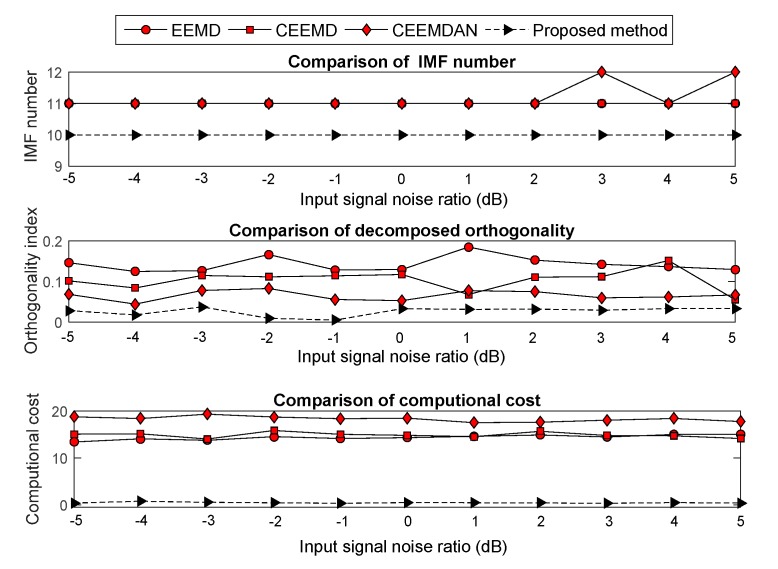
Comparison of the decomposition effect for the ‘heavy sine wave’ signal.

**Figure 10:**
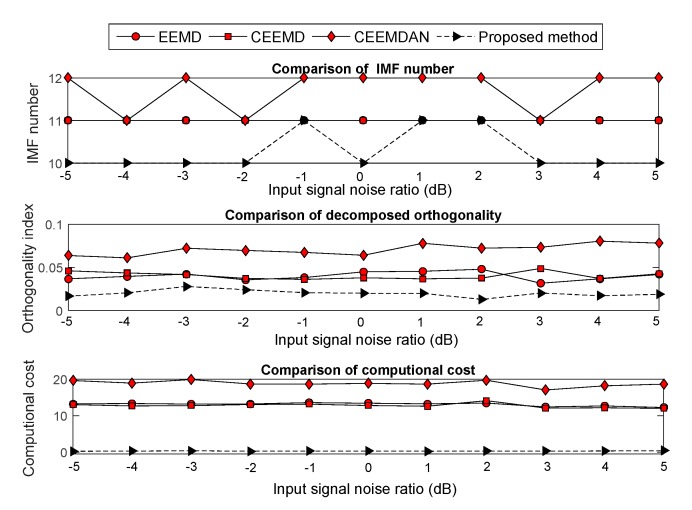
Comparison of the decomposition effect for the ‘quadchirp’ signal.

The Gaussian white noise, which had an input signal noise ratio (SNR) from −5 to 5 dB with step of 1 dB, was added into the four noisy signals. Moreover, the improved EMD versions were used to decompose the noisy signal. Evaluation methodologies, which include the computational cost, OI and decomposition number, were employed to analyze the decomposition effect under each SNR. The compared results are shown in Figure 7, Figure 8, Figure 9 and Figure 10. The results show that the computational cost and OI using ECEEMDAN is evidently smaller than that of the other methods, and the decomposing number is almost smaller than that of the improved EMD versions. These results indicate that the proposed method has a strong decomposition capability when the signal is subject to noise interference.

#### 3.3.3. Simulated Vibration Signal

In this section, ECEEMDAN is used to decompose the following simulated vibration signal (SVS):

(23) $$u\left(t\right)=G\sum_{i}u\left(t-iT\right)+n_{1}\left(t\right)$$

where G refers to the amplitude of the vibration signal, i refers to the number of oscillation periods of vibration signal, T refers to the period of vibration oscillation and reciprocal of characteristic frequency of bearing fault ($f_{c}$), and $n_{1}\left(t\right)$ refers to the noise component. u(⋅) is the operator of impulse response:

(24) $$u\left(t\right)=\left\{ \begin{array}{llll} \exp\left(-dt\right)\sin\left(2\pi f_{0}t\right) & & & t>0\\ 0 & & & t\leq 0 \end{array}\right.$$

where d refers to the attenuation constant and $f_{0}$ refers to the resonant frequency.

**Figure 11:**
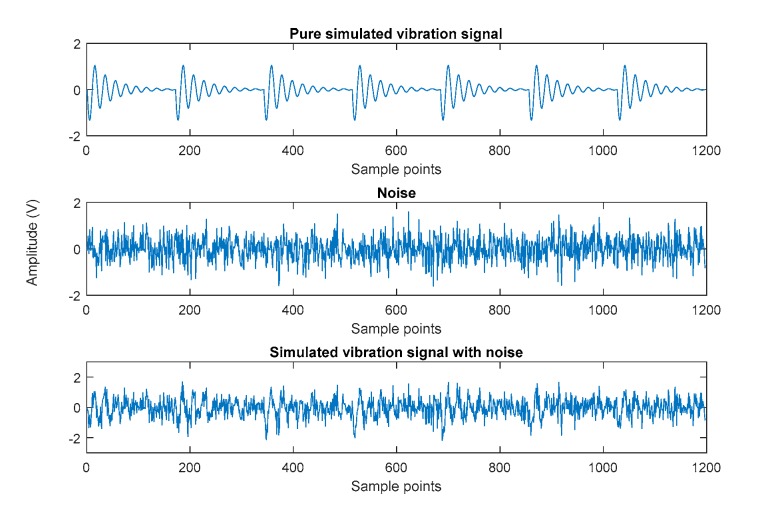
Simulated vibration signal.

The following parameters were used in the simulated signal: G at 1.4 $m/s^{2}$, d at 620, $f_{0}$ at 1 kHz, $f_{c}$ at 117 Hz, and a sample frequency ($f_{s}$) of 20 kHz. Here, the data length was 1197. White Gaussian noise, with SNR of 6 dB, was added to the simulated signal. Figure 11 presents the noise-free signal, the noise, and the noisy signal.

**Figure 12:**
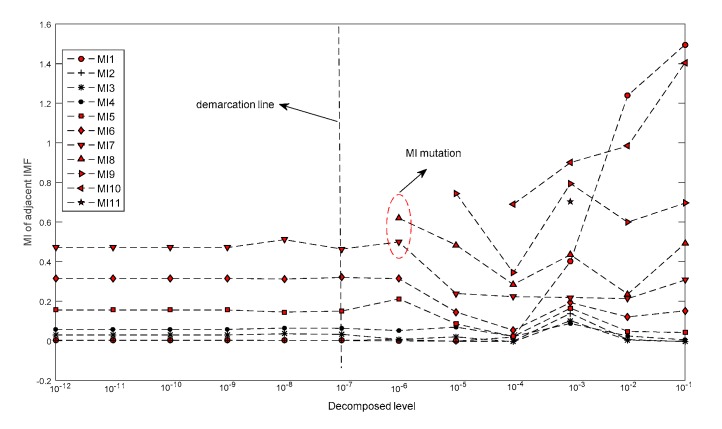
Decomposition result for simulated vibration signal (SVS) signal under different decomposing level.

The decomposition level was specified from 10^−12^ to 10^−1^, with a step of 10^1^. Figure 12 plots the MI of adjacent IMFs under each decomposition level, and Table 2 shows the decomposition number under each decomposition level. When the decomposition level was higher than 10^−7^, the MI demonstrated a marked change (shown as a red ellipse in Figure 12). The decomposition number changes from nine to ten, illustrating that the optimal decomposing level is smaller than 10^−7^. 

**Figure 13:**
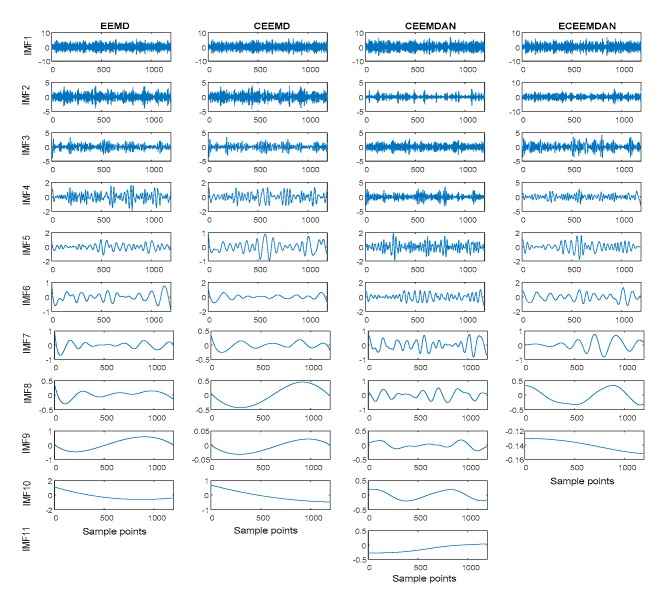
Decomposing result of SVS signal with the improved EMD versions and ECEEMDAN.

Moreover, the improved EMD versions were employed to decompose the SVS (ET and AAWN were 200 and 0.2, respectively). Figure 13 shows the decomposition result. The results indicate that the IMF numbers obtained using EEMD, CEEMD, and CEEMDAN were 10, 10, and 11, respectively, whereas that obtained using ECEEMDAN was only nine. These results illustrate that ECEEMDAN can restrain the spurious mode. 

Then, the OI and computational cost were utilized to compare the decomposition effect. Table 3 shows the compared results, which indicate that the computational cost and OI were smaller than those of the three others decomposition methods. This finding proves that the proposed method is more suitable for vibration signal decomposition than the traditional methods.

### 3.4. Identification of IIM 

Although ECEEMDAN can decompose a signal into a set of IMFs with physical meaning, each IMF does not completely and effectively reflect vibrational characteristics (intrinsic information). One of the IMFs mainly concentrates on the intrinsic information of the vibration signal. Traditionally, some statistical methods, such as the shape factor, crest factor, and impulse factor, as well as the kurtosis, skewness, and latitude factor, are used to identify IIMs. Table 4 shows the expressions of the statistical methods. However, these methods have different sensibilities and stabilities for various fault patterns. The latitude factor, impulse factor, and kurtosis are sensitive for initial issuance faults. Sensitivity significantly decreases a when fault is developed to a certain degree. The shape factor method is sensitive to fault features with low frequency. Kurtosis is suitable for diagnosing fault components from medium to high frequency. These findings indicate that a single method does not effectively identify IIMs when the fault pattern development is from slight to severe. Thus, this paper combines these statistical methods into the comprehensive factor (CF) to avoid the drawbacks of a single statistical method. Assuming that ECEEMDAN decomposes signals into a set of IMFs (IMF1, IMF2, …, IMFN). The normalized shape factor, crest factor, and impulse factor, as well as the kurtosis, skewness, and atitude factor can represented as Sh_*i*_, Cr_*i*_, Im_*i*_, Ku_*i*_, Sk_*i*_ and La_*i*_, respectively, for the *i*-th IMF. The CF can be expressed as:

(25) CFi=Shi∑i=1NShi+Cri∑i=1NCri+Imi∑i=1NImi+Kui∑i=1NKui+Ski∑i=1NSki+Lai∑i=1NLai

Here, the following selected criterion is used:

(26) k=arg max(CF)

where k is the index of the corresponding IIM. This is the adaptive threshold identified method. Table 5 shows the framework of the identified IIM.

**Figure 14:**
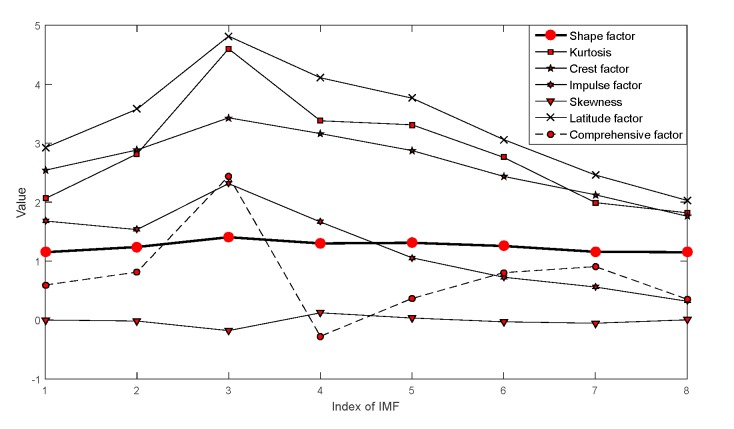
Comprehensive factor and factor of single statistical method.

**Figure 15:**
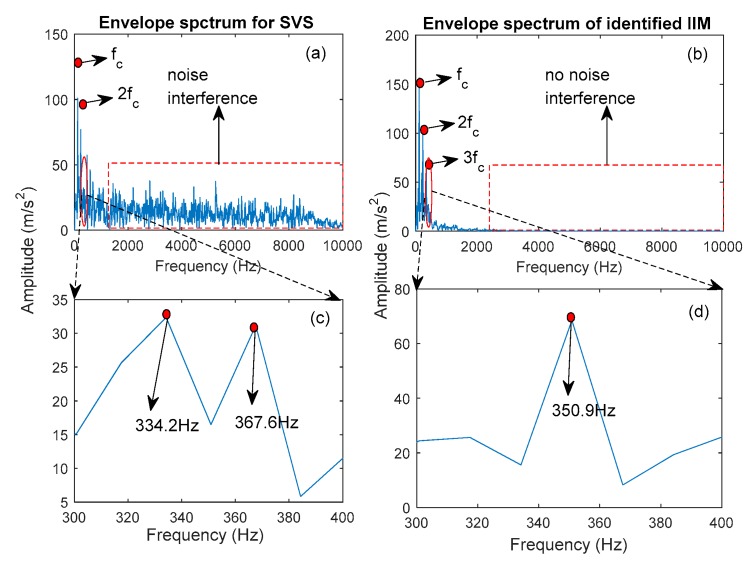
Comparison of fault frequency identification for the SVS signal and IIM. (**a**,**b**) the identified result of the feature frequency; (**c**,**d**) comprehensively shows the identified result.

**Figure 16:**
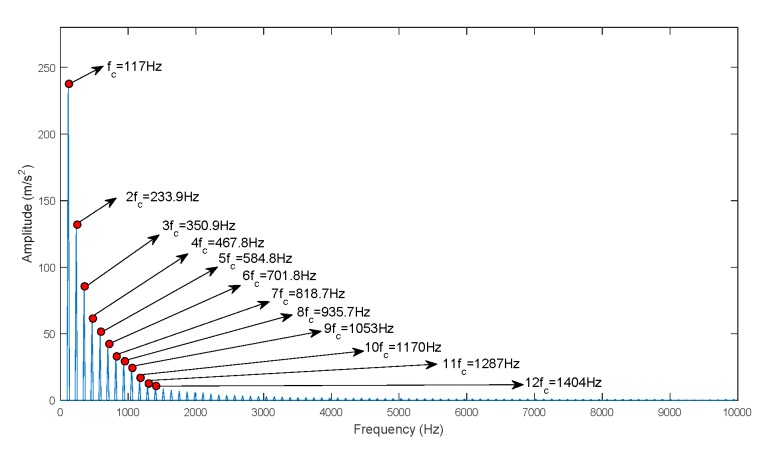
Octave frequency of the real signal.

**Figure 17:**
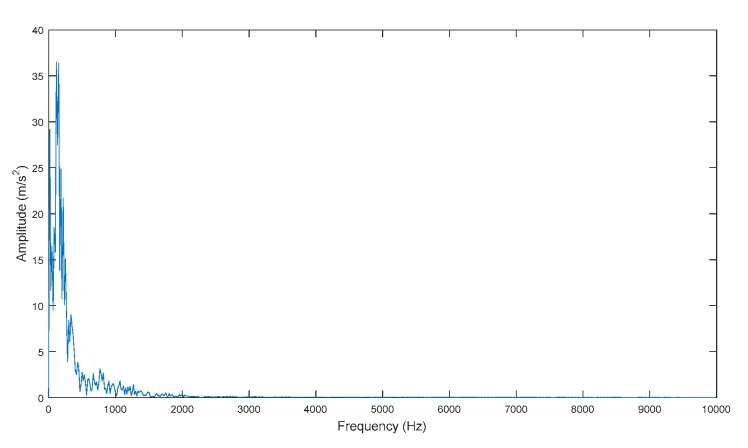
Envelope spectrum of the fourth IMF (IMF4).

In this study, the decomposed IMFs by ECEEMDAN in Figure 13 are used to select the IIM to prove the effectiveness of the CF. Figure 14 presents the CF and the single statistical method. The results show that the CF of IMF3 (CF_3_) is the largest. The envelope spectrum of IIM (IMF3) was determined to extract the feature frequency. Moreover, the original signal was analyzed to compare the identified effectiveness of feature frequency. Figure 15a,b shows the identified result of the feature frequency. Figure 15c,d comprehensively shows the identified result. The fault frequency ($f_{c}$) and second harmonic frequency ($2f_{c}$) are completely identified using the IIM and the original signal. However, reflecting the third harmonic frequency ($3f_{c}$) is difficult for the original signal, thus, $3f_{c}$ is reflected in the identified IIM. The peak frequencies were 334.2Hz and 367.6Hz for the original signal and 350.9Hz for IIM (here, $3f_{c}$ is consistent with the real signal, as shown in Figure 16 and Equation (23)). In addition, Figure 14 shows that the shape factor slightly changes and is unsuitable for IIM selection. The maximum value for the skewness factor is reflected in IMF4. The corresponding envelope spectrum for IMF4 is plotted in Figure 17. The result shows that $3f_{c}$ is difficult to identify.

The different input SNR and fault frequency (as shown in the input parameters in Table 6) were added to Equation (23) to further evaluate the discriminant capability of IIM. First, ECEEMDAN was used to decompose the SVS of corresponding cases, such as cases 1, 2, 3, 4, and 5 in Table 5. Then, the CF was used to identify the IIM for each case. Finally, the envelope spectrum of IIM was determined to identify the feature frequency. Meanwhile, the original signal is also used in the envelope spectrum to extract feature frequency and compare the identified effects. The identified result is shown in Table 7. The ‘yes’ means that the feature frequency can been correctly identified, whereas ‘no’ indicates the opposite. The fault frequency ($f_{c}$) and corresponding second harmonic frequency ($2f_{c}$) are both identified by IIM and the original signal. However, the third harmonic frequency ($3f_{c}$) can only be identified by the IIM. Thus, these results prove that the proposed CF method is suitable for the effective selection of IIM.

Thus, this paper proposes a novel identification method, namely ECEEMDAN with the addition of CF (ECEEMDAN-CF) for the diagnosis of fault features concerning rolling bearings:

(1) Obtain the IMF without interference of a spurious mode via ECEEMDAN.
(2) Find the maximum index of CF to obtain the IIM.
(3) Extract the fault feature from the rolling bearing via the envelope spectrum of the IIM.

**Figure 18:**
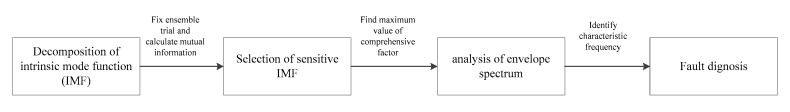
Flowchart of fault diagnosis.

Figure 18 shows the flowchart of fault diagnosis.

## 4. Performance Analysis

In this section, the proposed method (ECEEMDAN-CF) is used to extract fault features from rolling bearings. This method is comprised of two parts: (i) The data obtained from the Case Western Reserve University (CWRU) [35] and the (ii) real measurements from the experimental rig.

### 4.1. Experiment Data from the Case Western Reserve University

**Figure 19:**
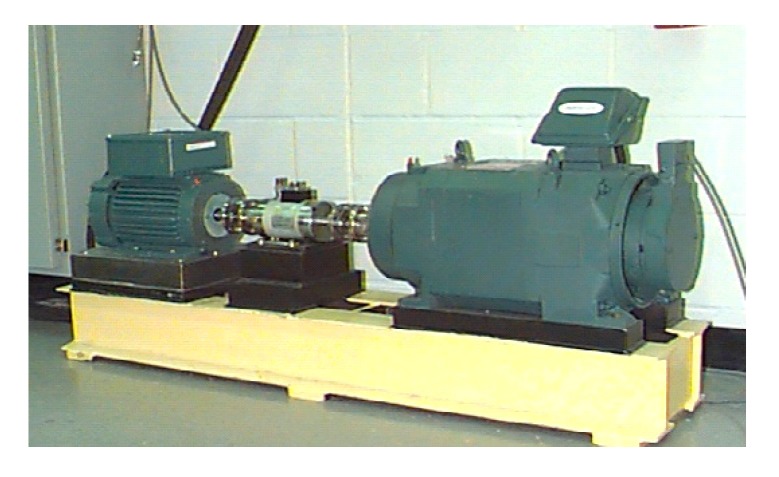
Test rig from the Case Western Reserve University.

The test rig was comprised of a 2 HP motor, a torque transducer, a dynamometer, and control electronics (Figure 19). The bearing type was 6205-2RS SKF, and the parameters are shown in Table 7.

**Figure 20:**
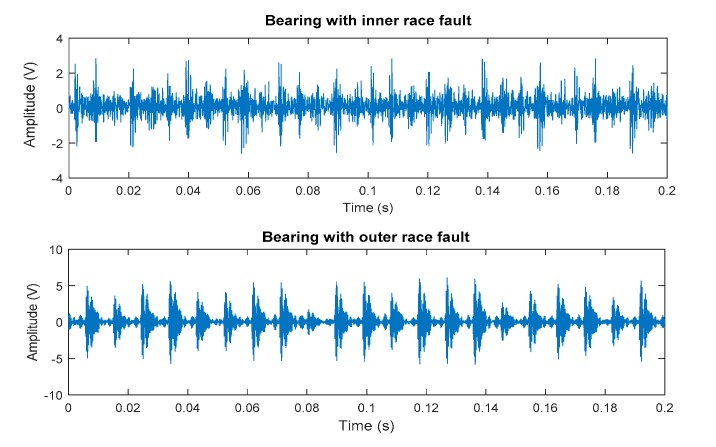
Experimental data of inner and outer raceway fault.

A single point fault was introduced into the test bearing by electrodischarge machining, with a fault diameter of 0.018 mm. The sample rate was 48 kHz. The rotation speed $f_{r}$ was 1797 RPM. The data from the inner and outer raceway fault were selected to extract the characteristic frequency (Figure 20) to evaluate the effectiveness of the proposed method. 

#### 4.4.1. Case 1 Bearing with an Inner Raceway Fault

The following expression is the theoretical characteristic frequency of the inner raceway fault ($f_{in}$):

(27) $$f_{in}=0.5n\left(1+\frac{d}{D}cos\;\alpha \right)f_{r}$$

**Figure 21:**
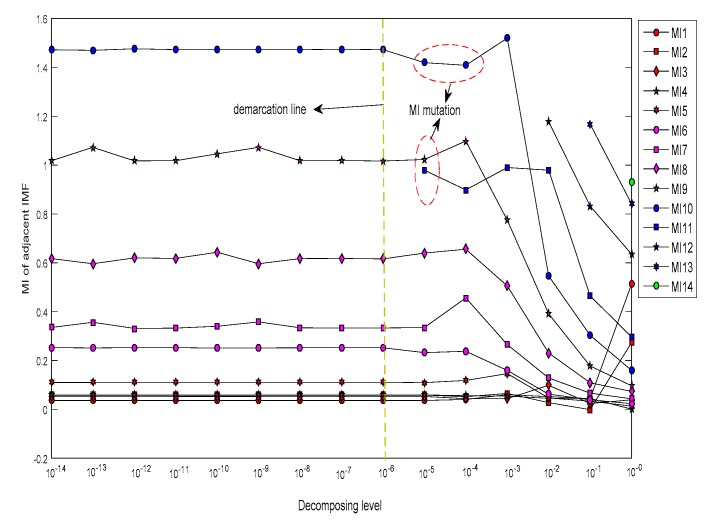
Decomposition result for a vibration signal with an inner raceway fault.

**Figure 22:**
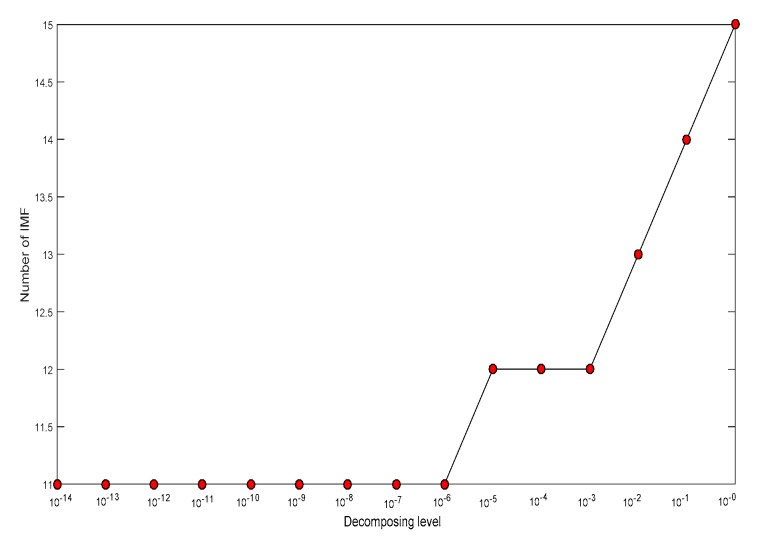
Decomposition number corresponding to each decomposition level.

First, ECEEMDAN is employed to decompose the fault signal. The decomposed level (L) is set from 10^−14^ to 10^0^, with a step of 10^1^, and the ET is fixed to two. The MI of the adjacent IMFs corresponding to each decomposition level are shown in Figure 21. The MI suddenly changed (shown as red ellipse in Figure 21) when the decomposed level was higher than 10^−6^, which is considered to be the optimal decomposition level. Figure 22 presents the decomposition number of the IMFs for each decomposition level.

**Figure 23:**
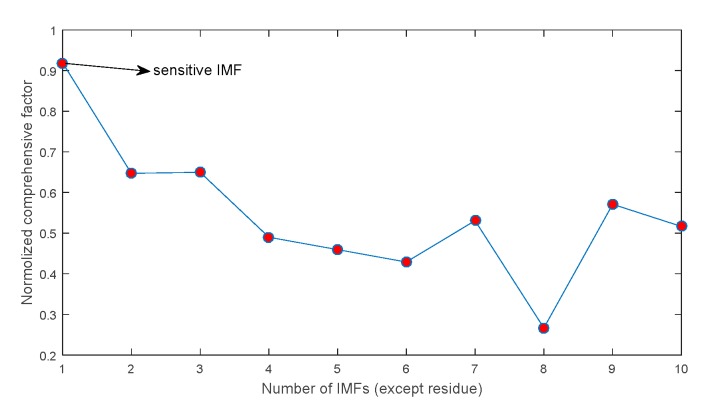
CF of each IMF.

**Figure 24:**
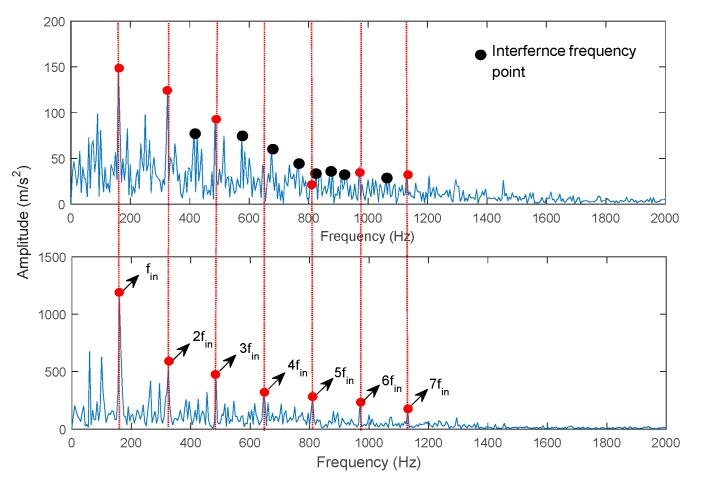
Comparison of extraction fault frequency for ECEEMDAN and CEEMDAN.

**Figure 25:**
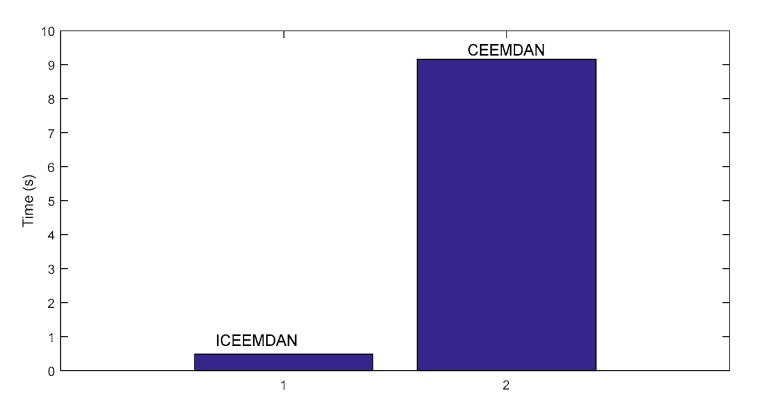
Comparison of computational cost for ECEEMDAN and CEEMDAN.

Figure 23 shows the CF of each IMF. Here, the first CF has the maximum value. This illustrates that the first IMF is the IIM. Meanwhile, the traditional CEEMDAN method was used to decompose the inner raceway fault signal, and the first IMF was selected to identify the envelope spectrum (Figure 24). The results show that the proposed method can identify the inner fault frequency and its corresponding harmonics (from second to seventh harmonics). The fault frequency is consistent with the theoretical frequency (Equation (26)). However, identifying the corresponding harmonics using CEEMDAN is difficult, because interference components disturb the extraction of fault information. Moreover, the computational cost is compared for the two methods. The computational costs of ECCEMDAN and CEEMDAN are 0.48 s and 9.16 s, respectively (Figure 25). Thus, the proposed method outperforms the traditional method in terms of fault frequency extraction for rolling bearings.

#### 4.4.2. Case 2 Bearing with an Outer Raceway Fault

**Figure 26:**
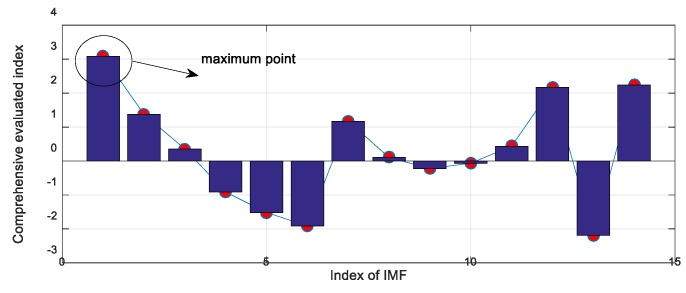
Selection of IIM with (ECEEMDAN-CF).

**Figure 27:**
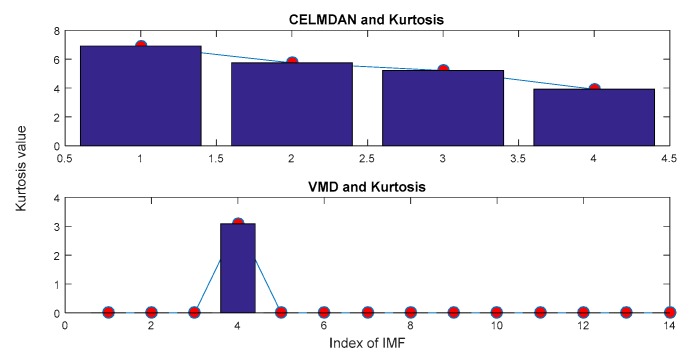
Selection of IIM with CELMDAN kurtosis and VMD kurtosis.

In this section, ECEEMDAN is used to decompose the fault signal. A total of 14 IMFs were obtained under the condition of the optimal decomposition level (10^−6^). Then, the CF was employed to identify the IIM (Figure 26). The results show that CF_1_ is the maximum, meaning that the first IMF contains the main information of the vibration signal. Meanwhile, complete ensemble local mean decomposition with adaptive noise (CELMDAN) -kurtosis and variational mode decomposition (VMD)-kurtosis [36] are also utilized to extract the IIM. Figure 27 shows the identified result of IIM for the two methods. Moreover, minimum entropy deconvolution (MED) [37] combined with autoregressive filter (AR) (MED-AR) was used to identify the fault frequency. The first and the forth IMF were the identified IIMs for CELMDAN kurtosis and VMD kurtosis, respectively.

**Figure 28:**
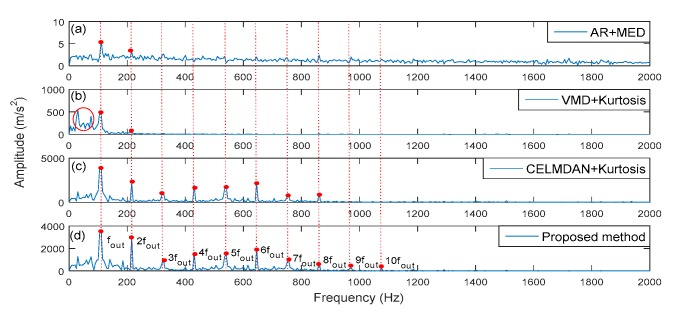
Comparison of fault extraction using the different methods.

Figure 28 shows the identified result of the fault frequency. It was found that the proposed method could identify the tenth fault harmonic (10$f_{out}$, shown as the red point in Figure 28d). Whereas, auto-regression- minimum entropy deconvolution (AR-MED), VMD kurtosis and CELMDAN kurtosis could only identify the second harmonics. From the results, more obvious disturbance components existed with the VMD kurtosis method than that with the proposed method (shown as red circle in Figure 28b). The amplitude energy of the fault characteristic for the AR-MED method was smaller than that of the proposed method (Figure 28a). This means that the proposed method had a better performance for the identification of fault features than the other methods.

### 4.2. Data from Real Measurement 

**Figure 29:**
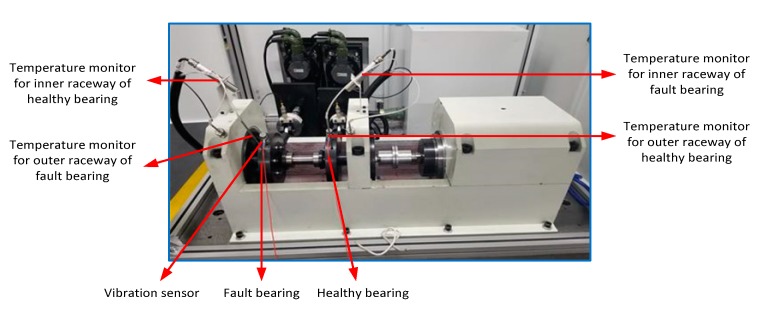
Experiment rig from real measurement.

In this section, the fault signal is acquired on the following test rigs (Figure 29). The defective bearing with an inner raceway fault and a healthy bearing were installed on the experiment rig. The temperatures of the inner and outer raceways were monitored by two wireless temperature sensors and two thermocouple sensors, respectively. Accelerometer sensors were installed on bearing house in the vertical direction. 

Below, Table 8 presents the bearing parameters.

**Figure 30:**
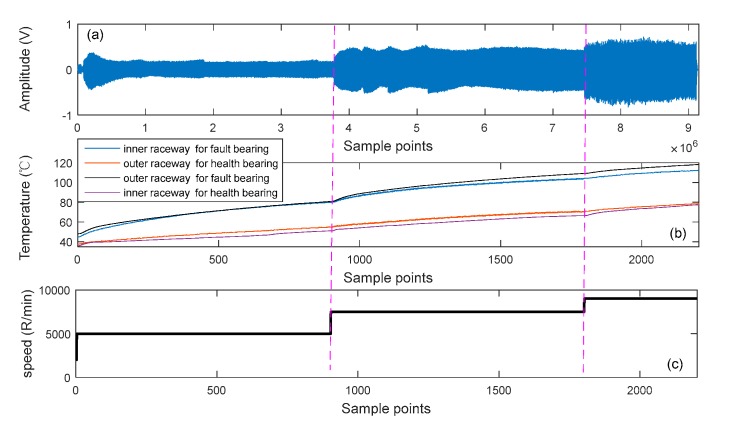
Monitored data of different test conditions. (**a**) vibration signal, (**b**) temperature signal, and (**c**) rotational speed.

The sample frequency ($f_{s}$) was 4 kHz. The rotating speeds of motor were set to 5000, 7500 and 9000 RPM. Figure 30 shows the acquired vibration signal, temperature signal, and rotational speed. At increased rotation speeds, the temperatures of the inner and outer raceways for fault bearing also increased, and the temperature of the outer raceway was higher than that of inner raceway for faulty and healthy bearings. 

Combining Table 8 and Equation (27), the theoretical feature frequency for faulty bearings under each test condition was 488.5 Hz at 5000 rpm, 732.7 Hz at 7500 rpm and 879.32 Hz at 9000 rpm. 

**Figure 31:**
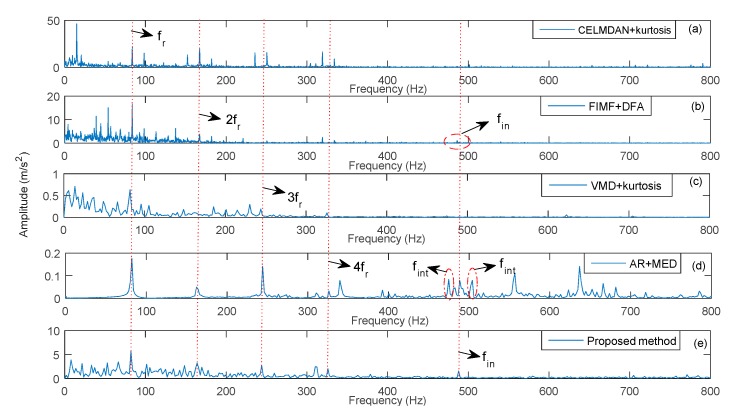
Identified fault frequency of a rolling bearing at 5000 RPM.

**Figure 32:**
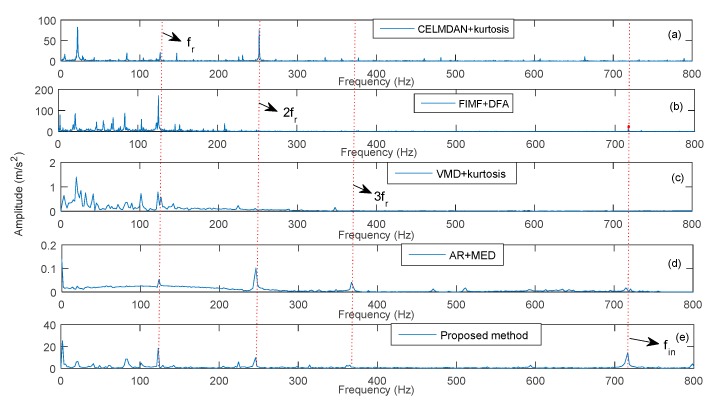
Identified fault frequency of a rolling bearing at 7500 RPM.

**Figure 33:**
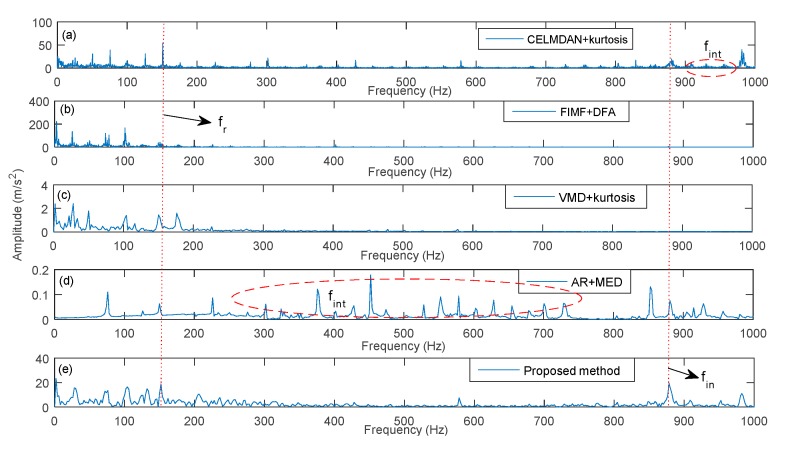
Identified fault frequency of a rolling bearing at 9000 RPM.

The proposed method was used to extract fault features under different rotation speeds. Meanwhile, the CELMDAN kurtosis, final intrinsic mode functions-de-trended fluctuation analysis (FIMF-DFA) [38], VMD kurtosis and AR-MED methods were used to extract the fault feature. The data length was 2048 for each test condition. Figure 31, Figure 32 and Figure 33 show the identified results at three different speeds. This finding indicates that the CELMDAN kurtosis and VMD kurtosis methods did not completely identify fault features for the three different test conditions. For the rotation speeds of 5000 and 9000 RPM, evident interference components ($f_{int}$) are observed around the fault frequency. When the rotation speed was 7500RPM, the amplitude of the fault frequency was considerably small, such that it was difficult to extract the fault frequency using the AR-MED and FIMF-DFA methods. The identified fault frequencies at 5000, 7500, and 9000 RPM, using the proposed method, were 488 Hz, 716.4 Hz and 878.4 Hz, respectively, with relative errors of 0.1%, 2.22% and 0.1%, respectively. Evidently, the fault frequency ($f_{in}$) can be identified by the proposed method, proving the suitability of the proposed method for fault component extraction under variable speed conditions. 

**Figure 34:**
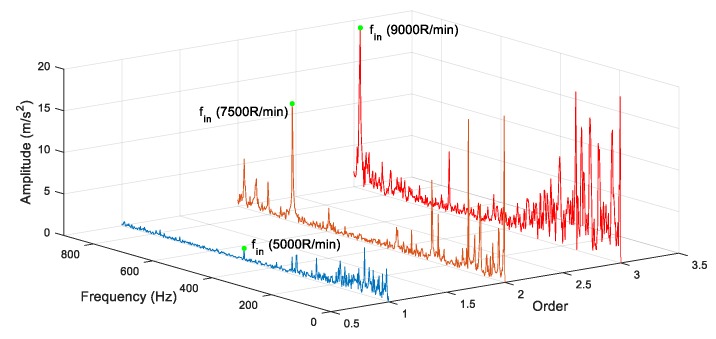
Comparison of feature frequency at different rotation speed.

Meanwhile, the feature frequency at different rotation speeds was also compared (Figure 34). The results show that a high rotation speed results in the large energy amplitude for the fault frequency.

## 5. Conclusions

This paper proposes a novel method for fault feature identification concerning rolling bearings. The following conclusions are drawn:

(1) The ECEEMDAN method, which sets ET as two, was introduced and used to determine the AAWN by the MI of adjacent IMFs under different decomposition levels. This method can improve the computational speed and avoid the interference of the spurious mode under interactions of added white noise and signal. ECEEMDAN also enhances the decomposition effectiveness of the original CEEMDAN method.
(2) The CF, which is established through combination with statistical methods, is proposed to identify the IIM of vibration signals. This method can compensate for the drawbacks of single statistical methods, such as insensitivity and unsteadiness, improving the identified effectiveness of the IIM.

In comparison with typical methods, the results indicate that the proposed method outperforms typical methods for the fault feature extraction of rolling bearings.

Notably, when the proposed method is used in various applications, the decomposition level should be investigated in future works.

## Figures and Tables

**Table 1:** Comparison of the decomposition effect.

Method	Number of IMF	Computation Time (s)	Orthogonality Index
EEMD	10	7.88	0.11
CEEMD	10	7.58	0.20
CEEMDAN	11	10.16	0.10
ECEEMDAN	4	0.09	0.03

**Table 2:** Decomposition number of the IMF under each decomposition level.

	Decomposition Level (L)											
	10^−12^	10^−11^	10^−10^	10^−9^	10^−8^	10^−7^	10^−6^	10^−5^	10^−4^	10^−3^	10^−2^	10^−1^
IMF number	9	9	9	9	9	9	10	11	12	13	12	12

**Table 3:** Comparison of decomposition effect for the simulation signal.

Method	Number of IMF	Computation Time (s)	Orthogonality Index
EEMD	10	17.83	0.15
CEEMD	10	16.59	0.14
CEEMDAN	11	25.89	0.12
ECEEMDAN	9	1.01	0.07

**Table 4:** Statistical methods of IIM selection.

Number	Statistical Parameter	Expression
1	Shape factor	$SH=\sqrt{\left(1/N\right)\sum_{i=1}^{N}x{\left(i\right)}^{2}}/\left(1/N\right)\sum_{i=1}^{N}\left|x\left(i\right)\right|$
2	Crest factor	$CR=max\left(\left|x\left(i\right)\right|\right)/\sqrt{\left(1/N\right)\sum_{i=1}^{N}x{\left(i\right)}^{2}}$
3	Impulse factor	$IM=max\left(\left|x\left(i\right)\right|\right)/\sqrt{\left(1/N\right)\sum_{i=1}^{N}\left|x\left(i\right)\right|}$
4	Kurtosis	$KU=\sum_{i=1}^{N}{\left(x\left(i\right)-\bar{x}\right)}^{4}/N{\left(x_{\sigma }\right)}^{4}$
5	Skewness	$SK=\sum_{i=1}^{N}{\left(x\left(i\right)-\bar{x}\right)}^{3}/N{\left(x_{\sigma }\right)}^{3}$
6	Latitude factor	$LA=N\;max\left(\left|x\left(i\right)\right|\right)/\sum_{i=1}^{N}\left|x\left(i\right)\right|$

Note: $\bar{x}$ and $x_{\sigma }$ refer to the mean and standard deviation, respectively, of time series x(i).

**Table 5:** Framework of identified IIM.

**Input**: A set of IMFs
Obtain number (N) of decomposing IMFs;
**For** i = 1:1:N
Calculate Sh_*i*_, Cr_*i*_, Im_*i*_, Ku_*i*_, Sk_*i*_ and La_*i*_ for *i*-th IMF (IMF_*i*_);
**End for**
Sum each statistical method: $\sum_{i=1}^{N}{\mathrm{Sh}}_{i}$,$\sum_{i=1}^{N}Cr_{i}$,∑i=1NImi,$\sum_{i=1}^{N}{\mathrm{Ku}}_{i}$,$\sum_{i=1}^{N}{\mathrm{Sk}}_{i}$ and $\sum_{i=1}^{N}{\mathrm{La}}_{i}$;
**For** i = 1:1:N
Calculate CFi=Shi∑i=1NShi+Cri∑i=1NCri+Imi∑i=1NImi+Kui∑i=1NKui+Ski∑i=1NSki+Lai∑i=1NLai;
**End for**
Find maximum of CF(${\mathrm{CF}}_{max}$);
Find index corresponding ${\mathrm{CF}}_{max}$: k=arg max(CF);
**Output**: the intrinsic information mode (${\mathrm{IMF}}_{k}$).

**Table 6:** Different simulation signals and corresponding identified result.

Cases	Input Parameters		Identified Results					
			Envelope Spectrum of Original Signal			Envelope Spectrum of IIM		
	SNR (dB)	Fault Frequency (Hz)	$f_{c}$	$2f_{c}$	$3f_{c}$	$f_{c}$	$2f_{c}$	$3f_{c}$
1	5	77	yes	yes	**no**	yes	yes	**yes**
2	4	87	yes	yes	**no**	yes	yes	**yes**
3	3	97	yes	yes	**no**	yes	yes	**yes**
4	2	107	yes	yes	**no**	yes	yes	**yes**
5	1	117	yes	yes	**no**	yes	yes	**yes**

**Table 7:** Bearing parameter of 6205-2RS deep groove ball bearing.

Bearing Type	Ball Number n	Pitch Diameter D (mm)	Ball Diameter d (mm)	Contact Angle α (°)
6205-2RS	9	52	8	0

**Table 8:** Bearing parameter in this experiment.

Ball Number n	Pitch Diameter D (mm)	Ball Diameter d (mm)	Contact Angle α (°)
10	46	7.932	0
